# The challenge of removing waste from wastewater: let technology use nature!

**DOI:** 10.1111/1751-7915.13711

**Published:** 2020-11-22

**Authors:** Olga C. Nunes

**Affiliations:** ^1^ LEPABE – Laboratory for Process Engineering, Environment, Biotechnology and Energy Faculdade de Engenharia Universidade do Porto Rua Dr. Roberto Frias Porto 4200‐465 Portugal

## Abstract

Tertiary treatments capable of removing chemical and biological contaminants of emerging concern have been successfully developed and implemented at full scale, opening the possibility of using wastewater treatment plants as recycling units, capable of producing wastewater that can be reused in various activities, such as agriculture irrigation; However, tertiary treatments remove only part of the wastewater microbiota, leaving the opportunity for regrowth and/or reactivation of potentially hazardous microorganisms, facilitated by the poor competition among the surviving microorganisms; Under the motto ‘added by technology, lead by nature’, the treatment and storage of treated wastewater must find the balance to develop a protection shield against the impoverishment the microbial quality and the development of potentially hazardous bacteria.

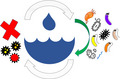

No man ever steps in the same river twice, for it's not the same river and he's not the same man. Heraclitus

Access to wholesome drinking water is not only a major ambition but also a basic human right that since antiquity has called scientists, engineers and politicians for action. The recognition that human excreta compromise the quality of the sources of drinking water triggered the development of sewage drainage systems as far as 3500–2500 BC, in cities such as Ur and Babylon (Lofrano and Brown, 2010). Among these ancient cities, Rome, where the largest known ancient sewer (Cloaca Maxima) and the first roman aqueduct (Aqua Appia) were built (600–312 BC), stands up (Lofrano and Brown, 2010). Despite the unexpected regression observed during the Middle Ages, the rising of urban and industrial agglomerations, matched by a growing production of wastewater, has been triggering the development of wastewater treatment technologies since the industrial revolution (Lofrano and Brown, 2010).

Unlike other industrial activities, whose high added value products enable high‐profit margins, wastewater treatment may be not prioritized, at least in world regions with limited income and capacity to invest in both infrastructure and operation systems. Consequently, most of the urban wastewater treatment plants (UWWTP) operating worldwide rely upon biological‐based low‐cost technologies. The conventional activated sludge (CAS) technology is one of the most commonly applied worldwide (Orhon, 2014). With a long development history itself, this aerobic biologic process, in full‐scale operation since 1914, is regarded as the conventional norm for wastewater treatment (Alleman and Prakasam, 1983; Orhon, 2014).

A century ago the major challenge of environmental engineers was to develop a treatment system able to reduce the load of readily degradable organic matter and pathogens from sewage. CAS‐based treatment systems fully achieve these goals (Tchobanoglous *et al*., 2003). But more than one century of industrial innovation and development changed dramatically our lifestyle, and consequently, the type of pollutants discharged in wastewater. Nowadays, UWWTPs are also expected to remove excess of inorganic nitrogen (N) and phosphorus (P) nutrients, responsible for the eutrophication of the receptor water bodies, and a myriad of (potentially) hazardous chemical micropollutants, which may pose risk to the aquatic ecosystems and human health given their acute and chronic toxicity. These chemical micropollutants of emerging concern, which are found at very low concentrations (up to μg l^−1^), include both natural and xenobiotic compounds such as pharmaceuticals, personal care products, steroid hormones, drugs of abuse, and pesticides, among others (European Commission, 2013; Ribeiro *et al*., 2015). In addition to the chemical micropollutants, UWWTPs are now also challenged to impede the release of high loads of biological contaminants of emerging concern, such as some pathogenic virus, protozoa, or bacteria in particular antibiotic‐resistant (ARB) harbouring antibiotic resistance genes (ARG), into the receptor water bodies (Dulio *et al*., 2018; European Commission, 2020).

Effective wastewater treatment systems are indeed the primary and major barrier between human activities and the environment, with a pivotal role on the prevention of contamination of surface‐ and groundwater. Inevitably, water bodies such as rivers, lakes and aquifers bridge sectors of activity and geographies, for instance when used as sources of agriculture irrigation water, drinking water production or habitat and fountain for wildlife or food‐producing animals. Pressures to implement technologies able to efficiently remove both chemical and biological contaminants within the urban water cycle are exacerbated under the climate change scenario. Massive withdrawal and consumption coupled with unpredictable weather conditions, such as drought and flood events, has been leading not only to freshwater scarcity but also to the deterioration of water quality (WWAP, 2019; European Commission, 2020).

Freshwater scarcity brought the new concept of UWWTPs as recycling units, capable of producing final effluents that can be safely and sustainably reused for different purposes, namely in agriculture, the sector with the largest consumption of freshwater (WWAP, 2019). But to be reused, treated wastewater must be safe. This means that the concentration of eventual chemical and/or biological pollutants in treated wastewater must not put at risk the environmental and human health. Hence, the degree of contamination of the treated wastewater determines its end use or site of discharge (European Commission, 1991, 2020; Becerra‐Castro *et al*., 2015).

Upgrading technologies capable of removal of N and P nutrients from wastewater have been successfully developed and implemented. Nowadays, full‐scale UWWTP with trains favouring the recirculation of the mixed liquor between aerobic and anoxic tanks, where ammonification of organic‐N, nitrification and denitrification occur according to the oxygen availability in each compartment are commonly found; and an increasing number of UWWTP where, in addition to the trains referred to above, recirculation includes anaerobic reactors favouring P granules accumulation are also operating worldwide (Tchobanoglous *et al*., 2003). More recently, the simultaneous C, N, and P removal is assured through the aerobic granular sludge technology, given the spatial distribution of the microorganisms of the different metabolic groups in the different micro‐environments of the granules (Nancharaiah and Reddy, 2018).

In contrast with the C, N and P removal, the biological removal of chemical micropollutants seems to be less efficient. Despite the ability of a vast number of microorganisms to degrade a wide diversity of micropollutants, the low concentration of these compounds in wastewater may contribute for their low bioavailability in the biological reactors. Consequently, the secondary final effluents of CAS‐based UWWTPs still contain numerous micropollutants at environmental worrisome concentrations (McEachran *et al*., 2018).

Advanced Oxidation Technologies (AOTs) have been recommended among the best solutions for the removal of chemical micropollutants from the secondary effluents of CAS‐based UWWTPs. A vast number of scientific studies has been conducted in this area, in order to develop and optimize tertiary processes capable of the efficient removal of these contaminants from the effluents before discharge into the receptor water bodies (Ribeiro *et al*., 2015). Among these technologies, ozonation has high visibility, being implemented in full‐scale UWWTPs, for instance in Switzerland, a country that recently implemented legislation recommending advanced treatment of wastewater aiming at protecting the environment (Rizzo *et al*., 2019).

One of the advantages of AOTs is their capacity to disinfect water (Rizzo *et al*., 2020). Hence, besides degrading undesirable chemical micropollutants, numerous scientific bench studies demonstrated that the mechanisms for microbial inactivation used by AOTs, such as the oxidative stress as it is generated by ozonation, are also capable of reducing the microbial load of wastewater, including ARB&ARGs (e.g. Rizzo *et al*., 2020). Such promising results opened the possibility of upgrading CAS‐based UWWTPs with a final AOT polishing step and using the facilities as recycling units of urban wastewater. Additional treatment may be required in a reuse scenario, and in that cases, the final treated wastewater may need to undergo an adsorption post‐AOT treatment step to eventually remove toxic degradation products (Rizzo *et al*., 2019) and to be stored for periods that may vary between few hours to some days, depending on the needs. Hence, some bench and full‐scale studies have been conducted to assess the microbiological quality of the wastewater after the final AOT treatment.

Consistently, studies focused on the effect of AOTs conclude that the microbiota, including ARB&ARGs, surviving AOT treatment is capable of re‐regrowth during the storage period, sometimes to values reaching or surpassing those measured in the untreated secondary effluent (Zimmermann *et al*., 2011; Becerra‐Castro *et al*., 2016; Czekalski *et al*., 2016; Sousa *et al*., 2017; Moreira *et al*., 2018; Biancullo *et al*., 2019; Iakovides *et al*., 2019). Moreover, re‐regrowth is accompanied by the disturbance of the microbial community, with possible implications on the decrease of diversity, and the overgrowth of *Proteobacteria* (Becerra‐Castro *et al*., 2016; Moreira *et al*., 2018). Among these, bacterial groups described as potential vectors of antibiotic resistance, such as *Pseudomonas*, have been detected at high relative abundance (Alexander *et al*., 2016; Jäger *et al*., 2018; Moreira *et al*., 2018).

The same phenomena occur when other technologies are applied in the wastewater treatment. Comparatively milder processes such as UV_254 nm_ irradiation or even coagulation lead to similar disturbances (Becerra‐Castro *et al*., 2016; Grehs *et al*., 2019). When comparing different technologies, a positive correlation between disinfection efficacy and the predominance of ubiquitous, potentially hazardous, bacteria in the treated stored wastewater seems to occur (Becerra‐Castro *et al*., 2016). Interestingly, clean built environments, where asepsis and frequent disinfection are the rule, are characterized by the predominance of *Proteobacteria* (Mahnert *et al*., 2019). Moreover, cleaning with aggressive agents seems to favour microbiomes encoding functions related with virulence, multi‐drug efflux, oxidative stress, as well as membrane transport and secretion, which empower cells to acquire nutrients in highly competitive nutrient‐poor environments (Mahnert *et al*., 2019).

Such results are not unexpected. Any process reducing the diversity and abundance of microorganisms in a given ecosystem, through physical removal of the cells or physical and/or chemical inactivation of macromolecules or cellular processes, is expected to generate a habitat where intercellular competition for space and nutrients is reduced, offering the opportunity for those that randomly survived the process and that are most versatile and fast to grow, to proliferate. Therefore, among the survivors, those with high capacity to grow under the conditions prevailing in the disinfected or cleaned system will thrive. Conversely, the microorganisms with specific requirements (e.g. nutritional) or with slower grow rates will be outcompeted.

*Proteobacteria* are well known for their genomic plasticity. Some proteobacterial species, such as *Pseudomonas aeruginosa*, colonize a wide diversity of environmental compartments, including mineral water, chlorinated drinking water, surface water and soils, and even human bodies (Grobe *et al*., 2001; Naze *et al*., 2010; Palleroni, 2015). Part of the success of this ubiquitous opportunistic pathogenic species rely upon its capacity to exchange genetic information through horizontal gene transfer (Kung *et al*., 2010). Hence, *Pseudomonas aeruginosa* harbour genetic information which allows cell development in a wide diversity of environmental conditions, including in the presence of a vast array of antimicrobial compounds. Therefore, besides carrying intrinsic antimicrobial resistance, *P. aeruginosa* strains are excellent vectors of ARG dissemination (Manaia, 2017). The predominance of microorganisms with these type of features in treated wastewater is thus not desirable, mainly if its further use in agriculture irrigation is envisaged, given the possibility of contamination of the food chain.

In this context, it may be argued that the upgrading UWWTPs with a final disinfection step is not enough to transform these facilities into wastewater recycling units, and more studies should be carried out to design and implement storage systems capable of attenuating the imbalance of the bacterial community before reuse of the stored treated wastewater.

Measures to restore the microbial richness and diversity of the disinfected wastewater would prevent the overgrowth of hazardous bacteria fitted to couple with very clean oligotrophic environments, such as *P. aeruginosa,* through competition. Such measures might include the inoculation of the disinfected wastewater with balanced natural microbial communities, with a rich and diverse phylogenetic and functional assembly of microorganisms (van Bruggen *et al*., 2019). In these communities, organisms belonging to a wide variety of species interact through complex relationships (mutualism, commensalism, competition, predation, parasitism) assuring metabolic redundancy and the integrity of nutrient cycles and energy flows (van Bruggen *et al*., 2019). Such communities are stable and resilient, that is, show little disturbance and restore rapidly upon alteration of the environmental conditions or invasion (van Bruggen *et al*., 2019). Hence, procedures such as diluting disinfected wastewater with non‐polluted surface water, mixing with pristine sediments or soils or discharge in wetlands would introduce a healthy microbiome in the treated wastewater. Under this circumstance, the exogenous microbiome would act as a protection shield for the proliferation of the hazardous microorganism surviving the disinfection process, in a similar way of the natural human microbiota, our first line of defence against the invasion of pathogens.

Definitely, microbes must have a say on removing waste from wastewater. The next research steps should be oriented towards a better understanding of the biotic relationships occurring in the treated wastewater and technological implementation of systems that are able to nurture these important artisan communities.
